# Operational Research during the Ebola Emergency

**DOI:** 10.3201/eid2307.161389

**Published:** 2017-07

**Authors:** Gabriel Fitzpatrick, Tom Decroo, Bertrand Draguez, Rosa Crestani, Axelle Ronsse, Rafael Van den Bergh, Michel Van Herp

**Affiliations:** Department of Public Health, Dublin, Ireland (G. Fitzpatrick);; Médecins Sans Frontières Operational Center Brussels, Brussels, Belgium (G. Fitzpatrick, T. Decroo, B. Draguez, R. Crestani, A. Ronsse, R. Van den Bergh, M. Van Herp)

**Keywords:** operational research, Ebola virus disease, Ebola virus, outbreak, Médecins Sans Frontières, infectious diseases, viruses, West Africa

## Abstract

Operational research aims to identify interventions, strategies, or tools that can enhance the quality, effectiveness, or coverage of programs where the research is taking place. Médecins Sans Frontières admitted ≈5,200 patients with confirmed Ebola virus disease during the Ebola outbreak in West Africa and from the beginning nested operational research within its emergency response. This research covered critical areas, such as understanding how the virus spreads, clinical trials, community perceptions, challenges within Ebola treatment centers, and negative effects on non-Ebola healthcare. Importantly, operational research questions were decided to a large extent by returning volunteers who had first-hand knowledge of the immediate issues facing teams in the field. Such a method is appropriate for an emergency medical organization. Many challenges were also identified while carrying out operational research across 3 different countries, including the basic need for collecting data in standardized format to enable comparison of findings among treatment centers.

Operational research is defined as the search for knowledge on interventions, strategies, or tools that can enhance the quality, effectiveness, or coverage of programs in which the research is being done (*1*). During the recent Ebola outbreak in West Africa, operational research was integrated into the response of Médecins Sans Frontières (MSF) to the emergency with the aim of controlling spread of the virus, improving patient outcomes, assessing the feasibility of new interventions, and advocating for policy change based on findings. Importantly, most operational research questions were decided by those in the field who had first-hand experience of the challenges encountered on a daily basis. This policy helped focus operational research to produce findings that were relevant to the emergency response.

MSF, in close collaboration with other actors such as the World Health Organization (WHO) and various national ministries of health, has been detecting and controlling Ebola outbreaks for decades and uses 6 pillars for its approach:

- Isolation of Ebola patients and supportive medical and mental health care in dedicated Ebola treatment centers (ETCs);
- Contact tracing;
- Raising awareness in the community;
- A functioning surveillance and alert system;
- Infection control in communities and ETCs:
- Maintaining healthcare for non-Ebola patients

ETCs operated by MSF admitted ≈5,200 patients with laboratory-confirmed Ebola virus infection, ≈2,500 of whom survived. Consequently, the organization was in the unique position of being able to use its data and experience to answer operational research questions that had an impact on the 6 pillars of the Ebola response. This article aims to summarize the key findings of this published operational research and identify lessons learned and knowledge gaps.

## Areas of Operational Research

### Understanding Patients and How the Virus Spreads

Operational research has provided detailed information on the clinical signs and symptoms of infected patients arriving at ETCs (*2*–*6*), which has allowed health professionals to anticipate what they should look for when assessing patients. In addition, the level of virus in the blood (viral load) was shown to be the strongest predictor of patient survival (*7*,*8*), and this measure was used to target care and counsel family members regarding expectations for their loved ones (*9*); a higher viral load represented a greater risk for death of the patient.

A single case of sexual transmission of Ebola virus was identified in early 2015 (*10*). During the outbreak, sexual transmission of Ebola virus probably accounted for only a small proportion of overall cases. However, this mode of transmission might also be responsible for the flare up of cases that occurred after the declaration of the end of the outbreak in Sierra Leone and Liberia. MSF has advocated the importance of not stigmatizing survivors of Ebola (based on risk for sexual transmission) because to do so would cause new Ebola patients to avoid seeking help (*11*).

Childbirth can also pose a risk for exposure in an Ebola setting. In one case of an Ebola virus–negative pregnant woman (who recently recovered), the fetus and surrounding amniotic fluid still harbored the virus (*12*). At delivery, there is considerable risk that those assisting in childbirth could be infected (*13*,*14*). Consequently, researchers have advocated that pregnant women who survived Ebola should be readmitted to the ETC when labor starts (*15*). The possible risk of Ebola passing from an infected mother to child through breast-feeding was also identified, resulting in a follow-up recommendation that breast-feeding be stopped and not restarted (*16*,*17*). The unexpected finding of a pregnant patient who initially was asymptomatic yet had a detectable Ebola viral load (*18*) showed how complex the study of Ebola virus transmission could be (*19*).

Psychologic counseling teams were employed throughout the outbreak to follow up and support the mental health of survivors. In Sierra Leone, an estimated one fifth of survivors were at high risk for developing posttraumatic stress disorder (*20*). These findings were used to advocate for more comprehensive care for survivors.

### Clinical Trials

Clinical trials, although not considered actual operational research, formed a substantial component of the research undertaken in the field. MSF, in partnership with other organizations, highlighted that a trial drug called Favipiravir was of some benefit to patients who arrived to ETCs with a low Ebola viral load (*21*,*22*). In a previous outbreak of Ebola, decades ago, convalescent-phase plasma from survivors was transfused into infected patients with inconclusive results. MSF, as part of a team of national and international organizations, launched the largest-ever trial of convalescent-phase plasma in Guinea. The interim results showed there was no significant increase in survival among those who received the plasma (*23*). Of note, the study’s findings are subject to some limitations and, consequently, further research is needed.

### The Community

MSF anthropologists had the important role of finding out what the affected communities thought of the Ebola virus and government control measures, such as quarantine and cremation. This research identified areas of misunderstanding and rumor within the community. Health promotion messages and outbreak control measures were then targeted to address these knowledge gaps. In addition, anthropologists were crucial for identifying the beliefs and behaviors within communities that facilitated further spread of the Ebola virus (*24*).

### Challenges within Ebola Treatment Centers

#### Laboratory Testing

The traditional way of diagnosing Ebola virus disease involved taking a blood sample from the patient by venipuncture and analyzing it by using PCR. At times, healthcare staff had difficulty performing venipuncture on very young children who were dehydrated (*25*). Staff performing venipuncture while wearing personal protective equipment (PPE) were also at risk for needle stick injuries that could result in nosocomial infections. Finger stick blood samples are much easier, faster, and safer to take than venipuncture samples. MSF questioned whether finger stick samples could be used instead of venipuncture for diagnosing Ebola virus disease. Staff in Guinea collected data on patients being screened for admission using both venipuncture and finger stick blood tests and found that finger stick samples were able to detect 87% of the Ebola cases confirmed using venipuncture samples (*25*). As a result of this research, it was recommended that finger stick blood sampling, although less accurate than venipuncture for diagnosing Ebola virus disease, could be used in situations where performing venipuncture was not possible.

The time between obtaining a blood sample and getting a PCR result can be considerable (*26*). MSF assessed the feasibility of using the Xpert Ebola Assay (Cepheid Inc., Sunnyvale, CA, USA) in the ETC setting and found that, when compared to traditional PCR testing, this assay reduced the waiting time between sampling and result notification by ≈50% (*26*). This difference was a major improvement in turnaround time for test results, especially for patients with suspected Ebola virus disease who were waiting to be admitted or discharged.

Different laboratories in the field used different types of PCR tests, which can give varying values for the viral load. As a result, comparing viral load results between laboratories for research purposes was occasionally difficult. MSF, in partnership with others, has advocated that standardized tests be employed so that viral load results can be compared among laboratory sites (*27*). Very rarely, the Ebola virus PCR test can give incorrect results; this fact was highlighted in an Ebola case from Monrovia with a false-negative PCR result (*28*). This complication underscores the need to always interpret test results in combination with the clinical and epidemiologic history of each patient.

#### Triage

Triage was used to determine which patients arriving to the ETC were likely to have Ebola virus infection or not. Patients who met the criteria of a suspect case using the WHO/MSF case definition were admitted to the suspect area of the ETC for blood testing. Patients whose illnesses did not meet the case definition were discharged from triage. If the triage step was not carried out appropriately with an accurate case definition, then some potentially infected persons could be sent home and noninfected persons could be admitted to the area reserved for patients with suspected Ebola virus infection. Such a scenario posed a threat for further spread of the virus (*29*). An accurate point-of-care test by which staff could determine without delay whether a patient has Ebola virus infection would greatly improve the triage process (*30*).

#### Infection Prevention and Control

The minimum level of PPE that is required when treating Ebola patients is still not fully understood. In general, the higher the level of PPE, the more difficult it is for staff to attend to patients in tropical environments because of heat stress and loss of dexterity. MSF participated with several organizations to explore what the optimal PPE should be, and this research is ongoing (*31*).

Infection control was critical for patients admitted from triage into the area of the ETC reserved for patients with suspected Ebola virus infection. While in this area, patients had phlebotomy performed to check for the presence of Ebola virus; a positive test result resulted in the patient being admitted to the confirmed ward and a negative test resulted in discharge. Therefore, patients with and without Ebola virus disease were gathered together in the same space at the same time. If infection control measures were inadequate, noninfected persons awaiting PCR results could contract the virus from infected persons also awaiting results. However, investigation found no evidence of this kind of spread occurring in ETCs (*32*).

## Effects of the Outbreak on Non-Ebola Healthcare

During the Ebola outbreak, researchers noted a major drop in clinic attendance by newly diagnosed HIV-positive patients and newly HIV-infected patients entering care in the affected countries (*33*). This trend was attributable to a combination of patients being afraid to attend clinics because of the known risk of contracting Ebola virus, clinics closing because of a lack of staff, and clinics reluctant to see new patients who might have symptoms compatible with Ebola virus disease.

Concerns were also raised that the control of tuberculosis (TB) in the region was jeopardized because of healthcare resources and personnel being focused solely on Ebola (*34*) and that operational research was required to document this problem and suggest strategies for better sustaining TB care during future epidemics. MSF’s experience in the affected countries showed that basic non-Ebola healthcare, including maternal (*35*) and child healthcare, was adversely affected during the epidemic. National ministries of health in the affected countries, supported by various organizations, are currently running operational research courses to study the impact of the outbreak on health systems.

## Evaluating Operational Research

The success of operational research is generally recognized to be measured within 4 domains (*36*). First, effective dissemination involves research findings being reported directly back to the field teams where the operational research was carried out. Second, the research should ideally be published in a peer-reviewed scientific journal. This step affords the work a level of acceptance within the wider community and can be a powerful aid when applied in the third domain, advocating for change to policy and practice. The fourth and most important measure of success is whether implementing the findings had a positive or negative impact on program performance and patient outcomes. Unfortunately, measuring impact is challenging and is often overlooked.

## Strengths of MSF Operational Research

Important findings from MSF operational research were disseminated back to the field in a timely and appropriate manner to maximize patient welfare. The operational research produced by MSF was mostly decided by returning field staff. These persons noticed particular operational issues while working in the field and then committed to carrying out research to address them on their return. Such a method for selecting research topics can make the results particularly relevant for field teams caring for patients but can also bias research output by only selecting topics that returning persons bring forward. To balance this, the operational research unit also supported the development of research questions that needed to be answered during the course of the outbreak. The combination of allowing both field and office staff to develop and carry out research created a productive environment for scientific inquiry.

The operational research output was prioritized with pressing field questions that could be answered by using routine program data fast-tracked for investigation (*25*). The priority areas aimed to reflect the 6 pillars of Ebola outbreak control, but this was not always possible. Collection of data relevant to some of the pillars was limited; therefore, operational research within these domains was not feasible. MSF advocated that research be published in open-access format to maximize readership. In addition, all operational research articles were placed on the organization’s field research website (http://fieldresearch.msf.org/msf).

## Challenges for Operational Research

Operational research requires the collection of accurate, harmonized, routine data, and one of the issues with the large number of MSF ETCs spread across 3 countries was that information was not collected in a standardized way. This discrepancy led to difficulties when trying to amalgamate and analyze patient data. Additionally, clinical interventions, such as the use of intravenous fluids, were not recorded systematically across ETCs. This lack of records proved to be a lost opportunity because retrospectively assessing what effect this intervention and others had on patient outcome was not possible. The use of personal digital assistants (i.e., small, mobile, handheld device that can store and retrieve information) has the potential to avert the problem of missed data collection in future outbreaks.

Research that was completed faced the challenge of finding journals that would review, accept, and publish the results in an appropriate timeframe. Some journals had the capacity to make quick decisions about publication, whereas others were slower and delayed the eventual dissemination of research to the wider scientific community. However, competing work priorities resulted in delays to some publications because authors were slow to finalize manuscripts.

The clinical trials were challenging to introduce in an emergency humanitarian setting and required impressive teamwork by all involved. Regrettably, even though the trials were fast-tracked compared with traditional timeframes, they were started very late in the outbreak, when case numbers were dwindling and efficacy was becoming more difficult to establish. In fact, many operational research questions across the 6 pillars of Ebola outbreak control still require comprehensive answers (Table).

**Table:** Operational research questions corresponding with the 6 pillars of Ebola outbreak control as observed by Médecins Sans Frontières*

Pillar of Ebola outbreak control	Key questions for operational research
Isolation of cases and supportive medical and mental health care in dedicated ETCs	Is it possible to provide isolation for case-patients outside the ETC setting, such as in the community?
Survivors of Ebola can suffer from physical and psychological side effects. How can their follow-up care be most effectively carried out?
Contact tracing	Which methods of contact tracing can provide the most comprehensive, relevant, and timely data in the field setting?
Raising awareness in the community	What novel methods of communications should be used for raising awareness and promoting health?
A functioning surveillance and alert system	How can communities be convinced to participate in the alert system in an effective manner?
Infection control in communities and ETCs	For how long does Ebola virus remain infectious in the environment of ETCs and the houses of case-patients?
Maintaining healthcare for non-Ebola patients	What is the efficacy, efficiency, safety, and feasibility of triage systems in non-Ebola health structures?

*ETC, Ebola treatment center.

## Future Directions for Operational Research

The need to collect continuous accurate routine program data must be fundamental for any future outbreaks. This collection of data should not just focus on clinical outcomes but must include all disciplines, such as water and sanitation and health promotion. In view of limited resources, the choice of operational research priorities for further investigation should be decided by a scientific committee of medical, operational, and external experts. This step would potentially avoid the introduction of bias when selecting operational research questions.

Similarly, a support team involving an editor, operational researcher, and statistician could greatly facilitate field staff translating their operational research questions and findings into peer-reviewed scientific publications. Consideration should be given to the creation of a web-based, open-access scientific journal for MSF that acts as a repository for all relevant operational research. Such a journal would avoid the delays encountered with the peer-review process among certain publications while also maintaining scientific standards.

MSF advocacy continues to support the development of internationally recognized protocols and ethical guidelines for clinical trials during emergencies so when the next emergency occurs, trials can commence much sooner. Several international organizations have already developed emergency preparedness plans that allow for the rapid activation of research and development activities during large-scale epidemics (*37*,*38*). The objective of these organizations is to expedite the availability of effective tests, vaccines, and medications that can be used to save lives and contain serious outbreaks. The future challenge is that, although different organizations can be united by the common humanitarian objective of stopping an outbreak, different partners might have different priorities and interests that can threaten collaboration.

## Conclusions

MSF has produced a comprehensive collection of published and unpublished operational research on the Ebola outbreak in West Africa. Importantly, the categories of research closely correspond to the 6 pillars of outbreak control described in this article. Operational research enables us to continually assess if new approaches are more effective than the ones currently in use and always aims to directly improve the care provided to those in need. The current model for deciding research topics within MSF is targeted toward addressing issues in the field. This approach is appropriate for an emergency medical organization. In this ever more connected world, MSF has advocated through its operational research for the creation of a functioning, international, rapid response capability for infectious disease outbreaks (*39*,*40*).
